# Older males exhibit greater dual-task gait variability and postural amplitude than older females

**DOI:** 10.1093/geroni/igaf122.4330

**Published:** 2025-12-31

**Authors:** Abeera Maham, Katherine Hsieh, Ashwini Pandey, Deborah Jehu

**Affiliations:** Augusta University, Augusta, Georgia, United States; Georgia State University, Atlanta, Georgia, United States; Augusta University, Augusta, Georgia, United States; Augusta University, Augusta, Georgia, United States

## Abstract

Greater dual-task cost (poorer cognitive and/or motor performance) heightens fall risk in older adults. There may be sex differences in dual-task cost that may inform tailored fall prevention strategies, but further research is needed. The purpose of this study was to examine whether sex differences influence dual-task cost during posture and gait in older males and females with poor mobility. A cross-sectional study was conducted in 85 older adults with self-reported poor mobility with no significant cognitive impairment (aged ≥65 years, 50 females; Montreal Cognitive Assessment scores>18). Using APDM wearable inertial sensors, participants stood with their feet apart and eyes open while completing no cognitive task, and while counting backwards by 3’s. Participants also walked at a comfortable walking speed while completing no cognitive task, and while naming words starting with “F”, “A”, or “S”. Two gait (step time coefficient of variability (CV), stride length CV, gait speed, double-support time, elevation CV) and postural (velocity, root mean square error (RMS), jerk, frequency) trials were completed. Males exhibited a greater step time CV during dual-task gait (F = 5.23, p=.009, males: 68.6±89.4%; females: 26.6±59.5%) and RMS during dual-task posture (F = 1.33, p=.009, males: 2884.5±1701.9m/s2; females: 2020.39±1458.19m/s2) than females in analyses of covariance, controlling for age and height. These findings suggest that greater variability in step time during gait and amplitude of postural sway in males relative to females serve as sensitive markers of dual-task cost in older adults. These results improve understanding of sex-specific differences in dual-task performance, guiding targeted interventions to enhance mobility.

